# Genetic predisposition to ischaemic stroke by *RAGE* and *HMGB1* gene variants in Chinese Han population

**DOI:** 10.18632/oncotarget.22112

**Published:** 2017-10-26

**Authors:** You Li, Jing Zhu, Linfa Chen, Weidong Hu, Mengxu Wang, Shengnan Li, Xuefeng Gu, Hua Tao, Bin Zhao, Guoda Ma, Keshen Li

**Affiliations:** ^1^ Guangdong Key Laboratory of Age-Related Cardiac and Cerebral Diseases, Affiliated Hospital of Guangdong Medical University, Zhanjiang 524001, China; ^2^ Department of Neurology, Affiliated Hospital of Guangdong Medical University, Zhanjiang 524001, China; ^3^ Institute of Neurology, Affiliated Hospital of Guangdong Medical University, Zhanjiang, Guangdong 524001, China

**Keywords:** *RAGE*, *HMGB1*, ischaemic stroke, gene variants, case-control study

## Abstract

Emerging evidence suggests that the multiligand receptor for advanced glycation end products (RAGE) and its ligand high mobility group box 1 protein (HMGB1) contribute to the pathophysiology of ischaemic stroke (IS). The present study aimed to investigate the association of *RAGE* and *HMGB1* variants with the risk of IS. A total of 1,034 patients and 1,015 age- and sex-matched healthy controls were genotyped to detect five genetic variants of the *RAGE* gene and four genetic variants of the *HMGB1* gene using the Multiplex SNaPshot assay. We found that the rs2070600 variant of *RAGE* was associated with an increased risk of IS (OR = 1.19, 95% CI: 1.02-1.38, P = 0.043), whereas the rs2249825 variant of *HMGB1* was associated with a decreased risk of IS (OR = 0.83, 95% CI: 0.71-0.98, P = 0.041). Further stratification by IS subtypes revealed that the presence of the TT genotype of the *RAGE* rs2070600 variant confers a higher risk of the large artery atherosclerosis subtype of IS (P = 0.036). Moreover, patients with the variant T allele of the *RAGE* rs2070600 variant presented with reduced serum soluble RAGE production. Patients carrying the variant G allele of the *HMGB1* rs2249825 variant exhibited significantly lower infarct volumes than those with the major CC genotype. These clues may help in the development of optimal personalized therapeutic approaches for IS patients.

## INTRODUCTION

Stroke is the leading cause of death and disability in China and other parts of the world [1]. Ischaemic stroke (IS) accounts for approximately 80% of all strokes and is caused by both genetic and environmental factors [2]. Although great endeavours have been made to elucidate the genetic make-up of IS, including genome-wide association studies [3, 4], the complete catalogue of genetic determinants is still unclear, which necessitates continuous exploration and precision in subsequent research.

The multiligand receptor for advanced glycation end products (RAGE), a member of the immunoglobulin superfamily, can interact with a broad range of ligands, including advanced glycation end products, high mobility group box-1 protein (HMGB1), S100/calgranulins and β-amyloid peptide [5]. HMGB1 is a nuclear protein with cytokine-type functions upon its extracellular release that are mediated by the activation of signalling pathways coupled to toll-like receptors (TLRs), including TLR4 and TLR2, both of which are involved in inflammatory responses [6-8]. The binding of RAGE to its ligands triggers the activation of reactive oxygen species, nuclear factor kappa-B, mitogen-activated protein kinase and protein kinase C; the up-regulation of leucocyte adhesion molecules; and the production of proinflammatory cytokines and angiogenic factors [9], all of which are responsible for the development and progression of IS. In contrast, the soluble form of RAGE (sRAGE) can potentially bind to an AGE ligand, thereby acting as a decoy and preventing the adverse effects of RAGE signalling [10].

A growing body of evidence indicates that RAGE signalling is implicated in the development and progression of various vascular diseases, including IS [11-14]. RAGE and HMGB1 are expressed in all cell types relevant to the development of atherosclerotic plaques, including endothelial cells, smooth muscle cells and monocytes/macrophages [9]. The expression of RAGE and HMGB1 was found to be significantly up-regulated in human atherosclerotic plaques and aortic vessels [15, 16]. *In vivo* evidence demonstrated that genetic ablation of RAGE reduced the infarct volume and abrogated macrophage activation in mice [17] and that the knockdown of HMGB1 expression by intra-striatal infection of *HMGB1*-shRNA reduced infarct size and microglia activation in experimental stroke models [18]. Moreover, clinical studies have also indicated that the plasma levels of RAGE and HMGB1 are significantly higher in subjects with IS [19, 20], whereas plasma levels of sRAGE are relatively lower [21]. These lines of evidence suggest that RAGE and its ligand HMGB1 may play a significant role in the pathogenesis of IS.

The genes encoding *RAGE* and *HMGB1* are highly polymorphic, and more than twenty single nucleotide polymorphisms (SNPs) have been investigated [22, 23]. Despite a large panel of *RAGE* and vascular disease-related genetic association studies, it remains unclear whether individuals possessing certain alleles are more susceptible to IS due their main or joint effects [24-27]. Therefore, we performed a case-control study to determine whether the variants of *RAGE* and *HMGB1* genes are associated with IS susceptibility in a relatively large southern Chinese population.

## RESULTS

### Demographic characteristics

The demographic characteristics of the participants are shown in Table 1. Of the 2,049 participants, 1,034 were patients with IS, and 1,015 were healthy controls. Smoking, diabetes, hypertension, drinking and hyperlipidaemia were significantly more common in the IS group than in the control group. There were no statistically significant differences between the patients and controls in terms of age or sex. The mean ages of the IS patients and controls were 68.33 years (± 10.90 years) and 67.57 years (± 9.12 years), respectively. The homocysteine (HCY) levels tended to be higher in the IS patients than in controls, whereas high-density lipoprotein (HDL) levels were lower at admission in the IS patients. The total cholesterol, triglyceride and low-density lipoprotein (LDL) levels in the IS patients were not significantly different from those in the healthy control subjects.

**Table 1:** Characteristics of ischaemic stroke cases and controls

Variables	IS (n=1034)	control (n=1015)	*P value*
Mean age (years)	68.33±10.90	67.57±9.12	0.120
Male/female	688 /346	666/349	0.659
Smokers, n (%)	261 (25.2%)	182 (17.9%)	**< 0.001**
Drinking, n (%)	101 (9.8%)	54 (5.3%)	**<0.001**
Hypertension, n (%)	663 (64.1%)	240 (23.6%)	< **0.001**
Diabetes, n (%)	266 (25.7%)	100 (9.9%)	< **0.001**
Hyperlipidaemia, n (%)	264 (25.5%)	197 (19.4%)	**0.001**
Total cholesterol (mg/dL)	5.05±1.13	5.12±1.07	0.225
Triglycerides (mmol/L)	1.55±0.92	1.51±0.99	0.360
HDL (mmol/L)	1.28±0.39	1.58±0.38	< **0.001**
LDL (mmol/L)	3.17±0.98	3.12±0.97	0.151
HCY (mmol/L)	12.88±4.52	10.34±2.80	<**0.001**

Continuous data are presented as the mean ± SD, median (range) or n (%).

^a^ P < 0.05 is indicated in bold font.

### *RAGE* and *HMGB1* gene variants and the risk of IS

The genotype and allele frequencies of the *RAGE* and *HMGB1* variants are shown in Table 2. No significant deviation from the Hardy-Weinberg equilibrium was observed for any of the nine variants compared to the genotype distributions of the healthy controls (data not shown), and their linkage patterns within each gene are illustrated in Supplementary Figure 2. Comparison of the genotype distributions between the IS patients and control subjects using the *χ*^2^ test revealed that there are statistical associations between the *RAGE* rs2070600 variant (P = 0.012) and the risk of IS. In a dominant model (CC vs. CT + TT), no significant difference was detected between the IS group and controls (P = 0.21). However, a significant difference in the frequency of the recessive model (CC+CT vs. TT) was observed in the IS group compared with the controls (OR = 2.14, 95% CI: 1.32–3.47, P = 0.008). The frequency of the T allele at the rs2070600 variant was significantly different in the IS group compared with the controls (OR = 1.19, 95% CI: 1.02–1.38, P = 0.043) after Benjamini-Hochberg (BH) multiple testing correction. In contrast, the genotype and allele distributions of the *HMGB1* rs2249825 variant differed significantly between the IS patients and the controls (P = 0.041). In a dominant model (CC vs CG+GG), no significant difference was detected between the IS group and controls (P = 0.11). However, a significant difference was observed in the frequency of the recessive model (CC+CG vs. GG) in the IS group compared with the controls (OR = 0.45, 95% CI: 0.24–0.84, P = 0.040). The frequency of the G allele at the rs2249825 variant was significantly different in the IS group compared with the controls (OR = 0.83, 95% CI: 0.71–0.98, P = 0.041) after BH multiple testing correction. We failed to find any statistical association between other variants and the risk of IS.

**Table 2:** Genotype and allele frequencies of *RAGE and HMGB1* variants between IS patients and controls, and corresponding ORs for IS

Genotype & Allele	IS patients (n=1034)	Controls (n=1015)	OR (95% CI)	*P value*	*P value*^*a*^
*RAGE*					
***rs1800624***					
TT	808(78.1)	768(75.7)		0.049	0.20
TA	192(18.6)	225(22.2)			
AA	34(3.3)	22(2.2)			
Dominant model TT vs TA+AA	226(21.9)	247(24.3)	0.87(0.71-1.07)	0.18	0.24
Recessive model TT +TA vs AA	1000(96.7)	993(97.8)	1.54 (0.89-2.64)	0.12	0.24
T allele	1808(87.4)	1761(86.7)	1.00		
A allele	260(12.6)	269(13.3)	0.94 (0.78-1.13)	0.52	0.52
**rs1800625**					
TT	890(86.1)	863(85.0)		0.73	0.73
TC	137(13.2)	143(14.1)			
CC	7(0.7)	9(0.9)			
Dominant model TT vs TC+CC	144(13.9)	152(15.0)	0.92(0.72-1.18)	0.50	0.73
Recessive model TT +TC vs CC	1027(99.3)	1006(99.1)	0.76(0.28-2.05)	0.59	0.73
T allele	1917(92.7)	1869(92.1)	1.000		
C allele	151(7.3)	161(7.9)	0.91(0.73-1.15)	0.45	0.73
**rs2070600**					
CC	655(63.3)	670(66.0)		0.006	**0.012**
CT	326(31.5)	320(31.5)			
TT	53(5.1)	25(2.5)			
Dominant model CC vs CT + TT	379(36.7)	345(34.0)	1.12 (0.94-1.35)	0.21	0.21
Recessive model CC + CT vs TT	981(94.9)	990(97.5)	2.14 (1.32-3.47)	0.002	**0.008**
C allele	1636(79.1)	1660(81.8)	1.000		
T allele	432(20.9)	370(18.2)	1.19 (1.02-1.38)	0.032	**0.043**
**rs1035798**					
GG	807(78.0)	768(75.7)		0.22	0.38
GA	210(20.3)	235(23.2)			
AA	17(1.6)	12(1.2)			
Dominant model GG vs GA + AA	227(22.0)	247(24.3)	0.88(0.71-1.07)	0.20	0.38
Recessive model GG + GA vs AA	1017(98.4)	1003(98.8)	1.40 (0.66-2.94)	0.38	0.38
G allele	1824(88.2)	1771(87.2)	1.000		
A allele	244(11.8)	259(12.8)	1.09(0.91-1.32)	0.35	0.38
**rs184003**					
GG	718(69.4)	674(66.4)		0.34	0.45
GT	288(27.9)	310(30.5)			
TT	28(2.7)	31(3.1)			
Dominant model GG vs GT+TT	316(30.6)	341(33.6)	0.87(0.72-1.05)	0.14	0.31
Recessive model GG+GT vs TT	1006(97.3)	984(96.9)	0.88(0.53-1.48)	0.64	0.64
G allele	1724(83.4)	1658(81.7)	1.000		
T allele	344(16.6)	372(18.3)	0.89 (0.76-1.05)	0.15	0.31
*HMGB1*					
**rs2249825**					
CC	736(71.2)	689(67.9)		0.021	**0.041**
CG	283(27.4)	294(29.0)			
GG	15(1.5)	32(3.2)			
Dominant model CC vs CG+GG	298(28.8)	326(32.1)	0.86(0.71-1.03)	0.11	0.11
Recessive model CC+CG vs GG	1019(98.5)	983(96.8)	0.45(0.24-0.84)	0.010	**0.040**
C allele	1755(84.9)	1672(82.4)	1.000		
G allele	313(15.1)	358(17.6)	0.83(0.71-0.98)	0.031	**0.041**
**rs1412125**					
TT	487(47.1)	451(44.4)		0.48	0.61
TC	448(43.3)	460(45.3)			
CC	99(9.6)	104(10.2)			
Dominant model TT vs TC+CC	547(52.9)	564(55.6)	0.90(0.76-1.07)	0.23	0.51
Recessive model TT+TC vs CC	935(90.4)	911(89.8)	0.93(0.69-1.24)	0.61	0.61
T allele	1422(68.8)	1362(67.1)	1.000		
C allele	646(31.2)	668(32.9)	0.93(0.81-1.06)	0.25	0.51
**rs3742305**					
GG	635(61.4)	592(58.3)		0.14	0.15
GC	357(34.5)	365(36.0)			
CC	42(4.1)	58(5.7)			
Dominant model GG vs GC+CC	399(38.6)	423(41.7)	0.88(0.74-1.05)	0.15	0.15
Recessive model GG+GC vs CC	992(95.9)	957(94.3)	0.70(0.47-1.05)	0.083	0.15
G allele	1627(78.7)	1549(76.3)	1.000		
C allele	441(21.3)	481(23.7)	0.87(0.75-1.01)	0.069	0.15
**rs1045411**					
GG	634(61.3)	590(58.1)		0.11	0.14
GA	358(34.6)	366(36.1)			
AA	42(4.1)	59(5.8)			
Dominant model GG vs GA+AA	400(38.7)	425(41.9)	0.88(0.73-1.05)	0.14	0.14
Recessive model GG+GA vs AA	992(95.9)	956(94.2)	0.69(0.46-1.03)	0.067	0.13
G allele	1626(78.6)	1546(76.2)	1.000		
A allele	442(21.4)	484(23.8)	0.87(0.75-1.01)	0.059	0.13

Data are presented as number (%).

^*a*^ adjusted for age, gender, smoking, hypertension, diabetes mellitus and hyperlipidaemia.

^b^ P < 0.05 is indicated in bold font.

### Haplotype analysis

Haplotype analysis of the *HMGB1* and *RAGE* genes was conducted separately because they are on different chromosomes, and the corresponding results are shown in Table 3. As low-penetrance haplotypes usually carry a high risk of producing false-positive findings, haplotype analysis was restricted to the common haplotypes, which had an estimated frequency of at least 3% in both patients and controls. In the *HMGB1* gene, the frequency of the A-C-G haplotype (corresponding to the rs1045411-rs3742305-rs2249825 variants) was significantly lower in IS patients than in controls (OR = 0.83, 95% CI: 0.70–0.98, P = 0.048) with a study power of 99.9%, and this haplotype was associated with a decreased risk of IS after adjusting for age, gender, smoking, hypertension, diabetes mellitus, and hyperlipidaemia when compared with the most common G-G-C haplotype. However, no significant associations were observed between the *RAGE* haplotypes and IS (Table 3). Multi-dimensionality reduction analysis did not reveal any significant gene-gene interaction models that were linked to IS risk (P > 0.05), as depicted in Supplementary Table 3.

**Table 3:** The frequencies of haplotypes of *RAGE* and *HMGB1* gene in patients and controls

Haplotypes	Case (n%)	Control (n%)	OR (95%)	*P value*	*P value* ^*^
*RAGE* (rs184003, rs1035798, rs2070600, rs1800624, rs1800625)					
G-G-C-T-T	879.0 (42.5)	858.0 (42.3)	1.00		
G-G-T-T-T	432.0 (20.9)	370.0 (18.2)	1.14(0.96-1.35)	0.13	0.47
T-G-C-T-T	344.0 (16.7)	372.0 (18.3)	0.90 (0.76-1.07)	0.25	0.47
G-A-C-A-T	243.0 (11.8)	259.0 (12.8)	0.92 (0.75-1.12)	0.39	0.47
G-G-C-T-C	151.0 (7.3)	161.0 (7.9)	0.92 (0.72-1.17)	0.47	0.47
*HMGB1* (rs1045411, rs3742305, rs2249825)					
G-G-C	1618 (78.6)	1539 (76.1)	1.00		
A-C-G	305 (14.8)	352 (17.4)	0.83 (0.70-0.98)	0.024	**0.048**
A-C-C	129 (6.3)	128 (6.3)	0.96 (0.74-1.24)	0.75	0.75

Adjusted for age, gender, smoking, hypertension, diabetes mellitus, and hyperlipidaemia.

^*^False discovery rate-adjusted P value for multiple hypotheses testing using the Benjamini-Hochberg method.

### Associations between *RAGE* or *HMGB1* gene variants and demographic characteristics

The associations between the *RAGE* rs2070600 and *HMGB1* rs2249825 gene variants and the demographic characteristics are shown in Table 4a. In an analysis stratified by gender, an increased risk of IS was associated with the variant genotypes CT and TT at the rs2070600 variant in female patients (P = 0.008 for the genotype, and P = 0.001 for the allele). In an analysis stratified by age, diabetes and hypertension, increased risk of IS was associated with the variant genotypes CT and TT at the rs2070600 variant in patients less than 70 years old (P = 0.012), nonhypertensive patients (P = 0.027) and nondiabetic patients (P = 0.027) (Table 4b).

**Table 4a:** A comparison between the baseline characteristics of the *RAGE* rs2070600 genotypes and alleles in the IS patient and control groups

Characteristics	IS patient group					Control group					*P*_*G*_^*a*^*value*	*P*_*A*_^*a*^*value*
	Genotype n (%)			Allele n (%)		Genotype n (%)			Allele n (%)			
	CC	CT	TT	C	T	CC	CT	TT	C	T		
Age												
≥70 years	327(63.2)	160(30.9)	30(5.8)	814(78.7)	220(21.3)	270(65.5)	124(30.1)	18(4.4)	664(80.6)	160(19.4)	0.562	0.370
<70 years	328(63.4)	166(32.1)	23(4.4)	822(79.5)	212(20.5)	400(66.3)	196(32.5)	7(1.2)	996(82.6)	210(17.4)	**0.012**	0.146
Gender												
Male	437(63.5)	219(31.8)	32(4.7)	1093(79.4)	283(20.6)	405(60.8)	246(36.9)	15(2.3)	1056(79.3)	276(20.7)	**0.027**	0.921
Female	218(63.0)	107(30.9)	21(6.1)	543(78.5)	149(21.5)	265(75.9)	74(21.2)	10(2.9)	604(86.5)	94(13.5)	**0.008**	**0.001**
Diabetes												
Yes	175(65.8)	77(28.9)	14(5.3)	427(80.3)	105(19.7)	69(69.0)	29(29.0)	2(2.0)	167(83.5)	33(16.5)	0.562	0.370
No	480(62.5)	249(32.4)	39(5.1)	1209(78.7)	327(21.3)	601(65.7)	291(31.8)	23(2.5)	1493(81.6)	337(18.4)	**0.027**	0.146
Hypertension												
Yes	422(63.7)	208(31.4)	33(5.0)	1052(79.3)	274(20.7)	167(69.6)	65(27.1)	8(3.3)	399(83.1)	81(16.9)	0.287	0.146
No	233(62.8)	118(31.8)	20(5.4)	584(78.7)	158(21.3)	503(64.9)	255(32.9)	17(2.2)	1261(81.4)	289(18.6)	**0.027**	0.214

P_G_: P value of the difference in alleles between the case and control groups; P_A_: P value of the difference in genotype between the case and control groups

^*a*^ adjusted for age, gender, smoking, hypertension, diabetes mellitus and hyperlipidaemia.

^b^ P < 0.05 is indicated in bold font.

**Table 4b:** A comparison between the baseline characteristics of the *HMGB1* rs2249825 genotypes and alleles in the IS patient and control groups

Characteristics	IS patient group					Control group					*P*_*G*_^*a*^*value*	*P*_*A*_^*a*^*value*
	Genotype n (%)			Allele n (%)		Genotype n (%)			Allele n (%)			
	CC	CG	GG	C	G	CC	CG	GG	C	G		
Age												
≥70 years	367(71.0)	144(27.9)	6(1.2)	878(84.9)	156(15.1)	272(66.0)	124(30.1)	16(3.9)	668(81.1)	156(18.9)	0.060	0.104
<70 years	369(71.4)	139(26.9)	9(1.7)	877(84.8)	157(15.2)	417(69.2)	170(28.2)	16(2.7)	1004(83.8)	202(16.7)	0.568	0.402
Gender												
Male	496(72.1)	181(26.3)	11(1.6)	1173(85.2)	203(14.8)	451(67.7)	196(29.4)	19(2.9)	1098(82.3)	234(17.7)	0.168	0.104
Female	240(69.4)	102(29.5)	4(1.2)	582(84.1)	110(15.9)	238(68.2)	98(28.1)	13(3.7)	574(82.2)	124(17.8)	0.168	0.402
Diabetes												
Yes	180(67.7)	83(31.2)	3(1.1)	443(83.3)	89(16.7)	70(70.0)	26(26.0)	4(4.0)	166(83.0)	34(17.0)	0.669	0.930
No	556(72.4)	200(26.0)	12(1.6)	1312(80.2)	224(19.8)	619(67.7)	268(29.3)	28(3.1)	1506(82.3)	324(17.7)	0.085	0.228
Hypertension												
Yes	468(70.6)	188(28.4)	7(1.1)	1124(84.8)	202(15.2)	154(64.2)	76(31.7)	10(4.2)	384(80.0)	96(20.0)	**0.040**	0.104
No	268(72.2)	95(25.6)	8(2.2)	631(85.0)	111(15.0)	535(69.0)	218(28.1)	22(2.8)	1288(83.1)	262(16.9)	0.568	0.381

P_G_: P value of the difference in alleles between the case and control groups; P_A_: P value of the difference in genotype between the case and control groups

^*a*^ adjusted for age, gender, smoking, hypertension, diabetes mellitus and hyperlipidaemia.

^b^ P < 0.05 is indicated in bold font.

Interestingly, when the patients were stratified based on hypertension, the decreased risk associated with the variant genotypes CG and GG at the rs2249825 variant was more significant in patients with IS than in controls (P = 0.004). However, when the patients were stratified by age, gender, or diabetes based on the *HMGB1* rs2249825 gene variant, no significant differences in the genotype or allele frequencies were detected between the IS patients and the controls (P > 0.05).

### Associations between *RAGE* and *HMGB1* gene variants and stroke subtypes

To explore whether the effects of *RAGE* and *HMGB1* gene variants are confined to a specific subtype or related to overall risk, we further separated the IS patient groups into stroke subgroups based on the CISS system [28]. The CISS system, which further classifies patients of minor stroke into known and precise aetiological categories, exhibits greater reliability for individual treatment and might therefore be more appropriate for use with Chinese patients with minor stroke [29]. As shown in Table 5, when the population was stratified according to the CISS classification system, the carriers of the TT allele at the *RAGE* rs2070600 variant appeared to have a higher risk of stroke of the large artery atherosclerosis (LAA) subtype compared with the controls (P = 0.036). No statistical associations were observed between the *HMGB1* rs2249825 variant and the stroke subtypes in the healthy controls.

**Table 5:** The relationship between *RAGE and HMGB1* genotypes and IS stratified by CISS classification in IS patients

	*RAGE* rs2070600							*HMGB1* rs2249825						
	Genotype			P value	Allele		P value	Genotype			P value	Allele		P value
	CC	CT	TT		C	T		CC	CG	GG		C	G	
Controls	670	320	25		1660	370		689	295	31		1673	357	
	(66.0)	(31.5)	(2.5)		(81.8)	(18.2)		(67.9)	(29.1)	(3.1)		(82.4)	(17.6)	
*Cases*														
LAA (n=653)	407	212	34	**0.036**	1026	280	0.088	460	182	11	0.279	1102	204	0.184
	(62.3)	(32.5)	(5.2)		(78.6)	(21.4)		(70.4)	(27.9)	(1.7)		(84.4)	(15.6)	
PAD (n=262)	173	79	10	0.468	425	99	0.839	190	68	4	0.279	448	76	0.184
	(66.0)	(30.2)	(3.8)		(81.1)	(18.9)		(72.5)	(26.0)	(1.5)		(85.5)	(14.5	
CS (n=46)	33	10	3	0.468	76	16	0.839	38	8	0	0.279	38	8	0.108
	(71.7)	(21.7)	(6.5)		(82.6)	(17.4)		(82.6)	(17.4)	(0.0)		(82.6)	(17.4)	
UE (n=70)	40	25	5	0.262	105	35	0.839	46	24	0	0.708	116	24	0.894
	(57.1)	(35.7)	(7.1)		(75.0)	(25.0)		(65.7)	(34.3)	(0.0)		(82.9)	(17.1)	

LAA: Large-artery atherosclerosis; PAD: Penetrating artery disease; CS: Cardioembolic Stroke; UE: Undetermined aetiology.

### The serum levels of sRAGE based on the *RAGE* rs2070600 and *HMGB1* rs2249825 genotypes

**Figure 1:**
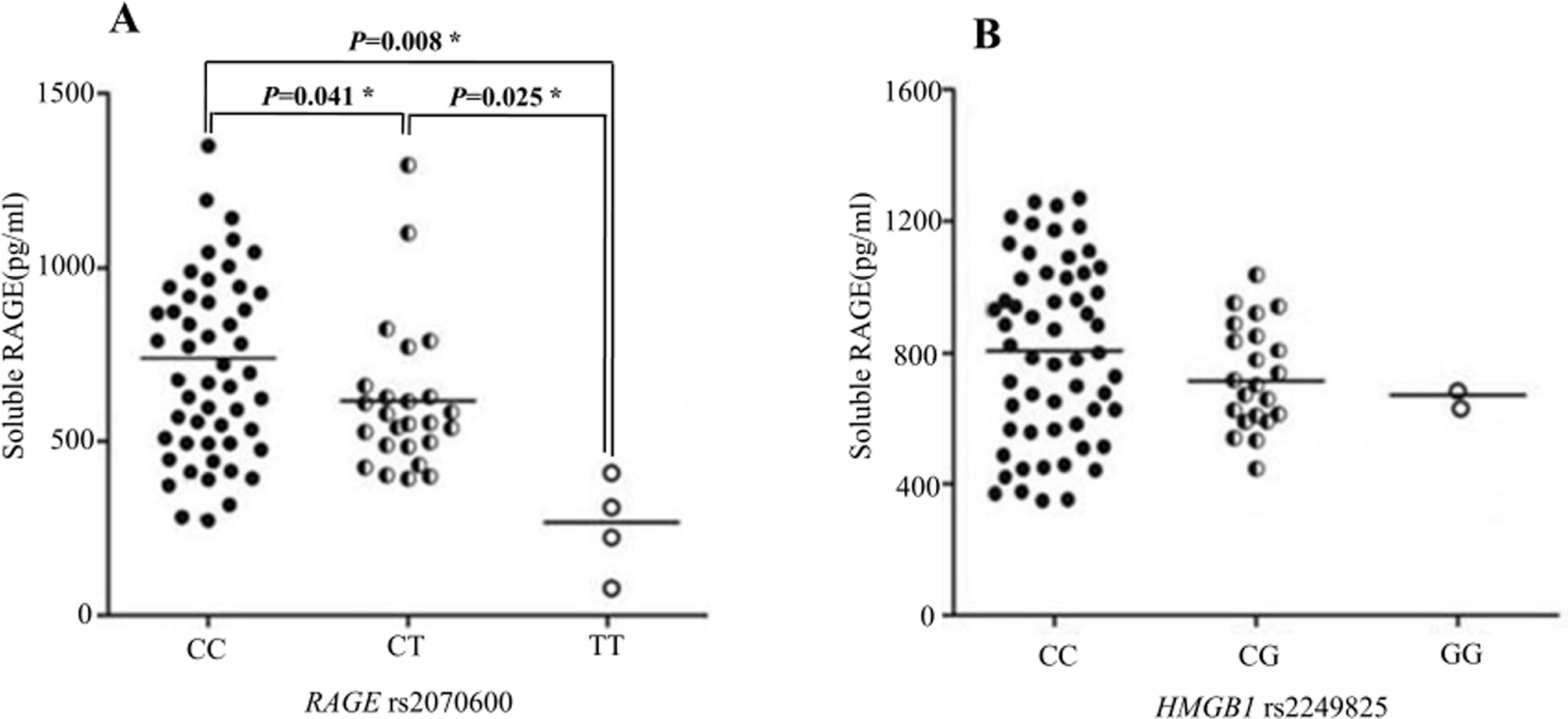
The serum sRAGE levels in IS patients stratified based on *RAGE* genotype **(A)** and *HMGB1* genotype **(B)**. The serum sRAGE levels in IS patients were measured using ELISA. The data are shown as the mean ± SD. Comparisons of the serum sRAGE levels among patients with different RAGE and *HMGB1* variants in the case and control groups were evaluated using Student’s *t*-test for normally distributed data, and for non-normally distributed data, a Mann-Whitney U nonparametric test was used. An asterisk indicates P < 0.05.

The serum levels of sRAGE measured in 84 IS patients are presented in Figure 1. When patients were stratified based on *RAGE* genotype, the serum sRAGE levels were significantly lower in patients with the CT and TT genotypes at the rs2070600 variant than in those with the CC or CT genotypes at the rs2070600 variant (P = 0.041 and P = 0.008, respectively) (Figure 1A). However, when the patients were stratified based on *HMGB1* genotype, no significant differences in serum sRAGE levels were detected among IS patients with different genotypes (Figure 1B).

### Association of the *RAGE* rs2070600 variant with the infarct volume

**Figure 2:**
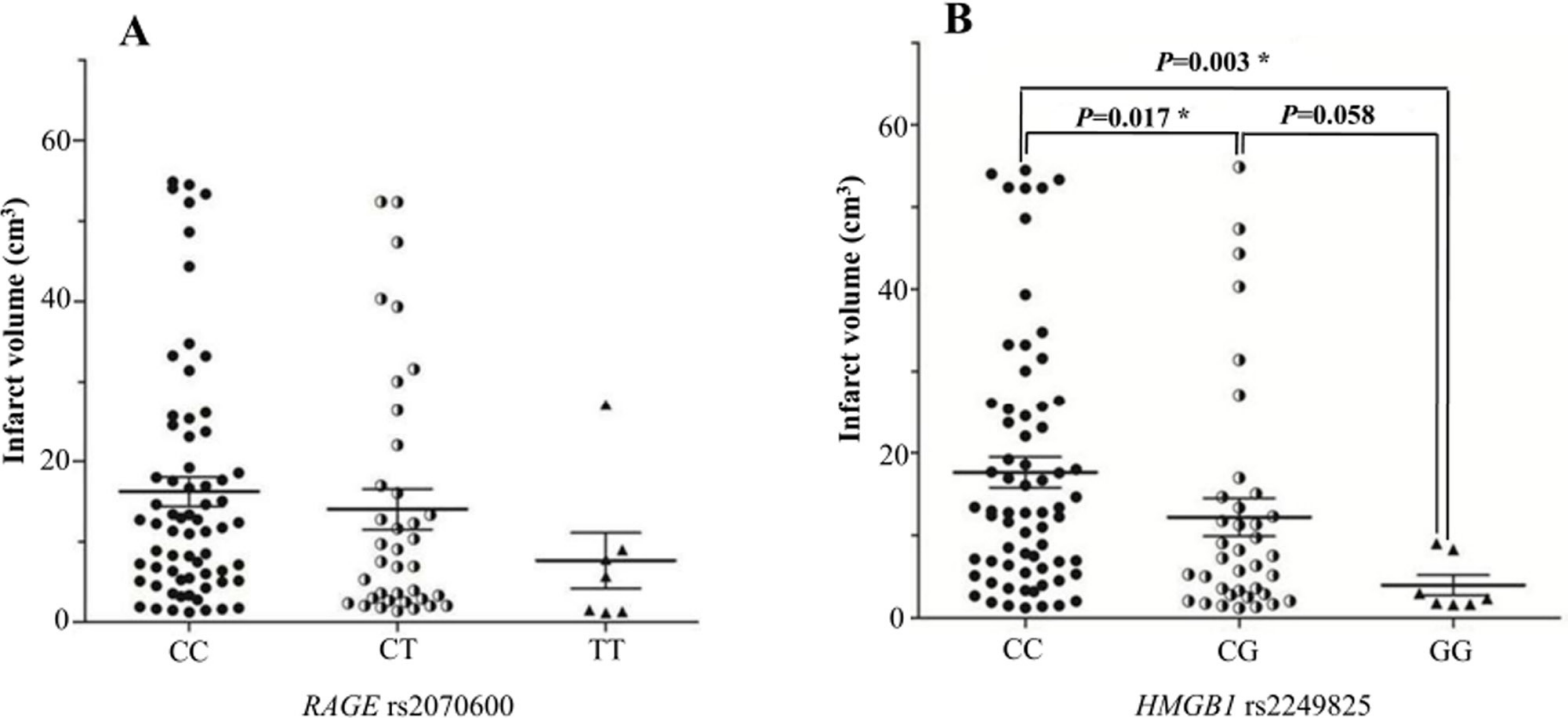
The mean infarct volumes ± SD in the IS patients stratified based on *RAGE* genotype **(A)** and *HMGB1* genotype **(B)**. The data are shown as the mean ± SD. Correlations between the genotypes of the *RAGE* and *HMGB1* variants and the DWI infarct volume were assessed using ANOVA. An asterisk indicates P < 0.05.

Associations of the *RAGE* and *HMGB1* polymorphisms with the actual volumetric measurements of infarct volume by DWI in 111 IS patients were explored, and the results are shown in Figure 2. The mean infarct volumes in the IS patients with the *HMGB1* variant genotypes (CG and GG) were significantly lower than those in patients with the major CC genotype (P = 0.017 and P = 0.003, respectively). However, no difference in infarct volume was observed in IS patients with the *RAGE* variant genotypes or major genotypes.

## DISCUSSION

In this hospital-based, case-control study, we examined whether nine well-defined variants in the *HMGB1/RAGE* pathway genetically predisposed individuals to IS in a relatively large southern Chinese population, and we observed a significant association between the *RAGE* rs2070600 and *HMGB1* rs2249825 variants and the risk of IS. The TT carriers at the *RAGE* rs2070600 variant had a significantly increased risk of IS, whereas individuals carrying the variant genotypes (CG and GG) of the *HMGB1* rs2249825 variant had a decreased risk of IS. Haplotype analysis suggested that the *HMGB1* A-C-G haplotype (corresponding to the rs1045411-rs3742305-rs2249825 variants) exhibited a protective effect against IS susceptibility. Further stratification revealed that the variant TT allele of the *RAGE* rs2070600 variant is associated with a higher risk of IS of the LAA subtype. The serum sRAGE levels were significantly lower in IS patients with the variant CT and TT alleles of the *RAGE* rs2070600 variant than in those carrying the major CC genotype. Additionally, patients carrying the variant genotypes (CG and GG) of the *HMGB1* rs2249825 variant had lower infarct volumes than those with the major CC genotype.

The three most extensively studied functional variants of the *RAGE* gene include two SNPs in the promoter region (-429T/C and -374T/A) and one SNP in exon 3 (G82S). The rs1800625 (-429T/C) and rs1800624 (-374T/A) polymorphisms of *RAGE* have been shown to exert significant effects on transcriptional activity [30], and the rs2070600 (G82S) polymorphism, which is localized in the N-linked glycation site, has been shown to enhance ligand binding affinity and lead to increased ligand-stimulated activation of proinflammatory mediators [22]. To date, several studies have examined the association between the *RAGE* polymorphisms and susceptibility to IS. Zee *et al.* reported that the T-A-G haplotype of the (rs1800625-rs1800624-rs2070600) *RAGE* gene polymorphisms was associated with reduced risk of IS in Caucasians [25]. Olsson *et al.* found that one polymorphism of the *RAGE* gene at rs1035798 was significantly associated with a subtype of small-vessel disease but not overall IS in the Caucasian population [24]. Cui *et al.* found that the rs2070600 polymorphism of the *RAGE* gene was associated with increased risk for overall IS in the Chinese Han population [26]. In our case-control study, we observed that the mutant T allele of the rs2070600 variant in the *RAGE* gene was significantly associated with an increased risk of developing IS; this result is consistent with Cui’s report. However, a cautionary note should be mentioned concerning the difference in the mutant allele frequency of the rs2070600 variant in controls. In our population, the rs2070600 allele frequency among the controls was 18.2% for the T allele, which is significantly lower than the rs2070600T allele frequency in individuals from Cui’s study (45%). In addition, the prevalence of the rs2070600T allele in the controls of our study is similar to that in the HapMap database for the CHB population (https://www.ncbi.nlm.nih.gov/variation/tools/1000genomes/). This discrepancy might result from profound ethnic and geographic differences. In addition, differences in sample size, patient selection criteria and research strategy in these studies may also explain this difference.

Several previous studies have examined the association between *HMGB1* polymorphisms and vascular diseases. Qu *et al.* found that the G allele of the *HMGB1* rs2249825 polymorphism was associated with an increased risk of postoperative atrial fibrillation after cardiac surgery [31]. Yao *et al.* suggested that the rs2249825 *HMGB1* polymorphism is significantly associated with pulmonary hypertension and diastolic blood pressure [32]. A very recent study indicated that the minor G allele of the rs2249825 polymorphism was associated with an increased risk of delayed cerebral ischaemia after aneurysmal subarachnoid haemorrhage [33]. In the present study, we reported for the first time that the G allele of the *HMGB1* rs2249825 variant was associated with protection against IS. Furthermore, the A-C-G haplotype (corresponding to the rs1045411, rs3742305 and rs2249825 variants) was associated with a 0.83-fold decreased risk of developing IS. Bioinformatics analysis showed that the variation of C to G at the rs2249825 SNP may affect transcription factor v-Myb binding to the *HMGB1* binding sites [33] (FASTSNP, http://fastsnp.ibms.sinica.edu.tw), thereby influencing the regulation of HMGB1 expression. Zeng *et al.* reported that the rs2249825 SNP may be associated with HMGB1 production in peripheral blood leukocytes [34]. This evidence supports the view that *HMGB1* rs2249825 variants could affect its binding with v-Myb, thereby influencing the regulation of HMGB1 expression. Although increasing numbers of studies have investigated the associations of variants in the *HMGB1/RAGE* axis with susceptibility to stroke, the results have been inconsistent. Buraczynska *et al.* found that the A allele of the -374 T/A polymorphism in the *RAGE* gene has a protective effect against stroke [35]. Cui *et al.* reported that the haplotype C_-429_G_82_T_-374_of RAGE showed a protective effect against IS susceptibility [26]. These lines of evidence and our findings collectively indicate that variants in the *HMGB1/RAGE* axis may play protective roles in stroke development. Additionally, HMGB1 plays a biphasic role in inflammation and stroke [36]. HMGB1 amplifies the inflammatory response during acute ischaemic injury [18]; nevertheless, HMGB1 may also improve endothelial activation [37, 38] and enhance neurite outgrowth as well as neuronal survival [39, 40]. Our observation that carriers of the G allele of the rs2249825 *HMGB1* variant have a decreased risk of IS may have resulted from the beneficial role of HMGB1 in IS.

sRAGE is a C-truncated secretory isoform of the receptor protein and functions as a decoy for the cell surface receptor, thus blocking cell activation by RAGE and inhibiting the chronic inflammation, diabetes, atherosclerosis and metabolic syndrome triggered by RAGE signalling [9, 41, 42]. Therefore, the release of sRAGE may be a potential protective factor for atherosclerosis [43]. The glycine-to-serine change at position 82 occurs proximal to an N-linked glycosylation site (position 81) and within the ligand-binding V-domain of RAGE; this variant may affect RAGE function [44]. Gaens *et al.* showed that the S allele of the G82S *RAGE* gene polymorphism is strongly associated with lower sRAGE levels in Caucasians, which may be explained by the higher binding affinity of RAGE for ligands caused by the N-linked glycosylation state of the protein [22]. In the present study, we found that individuals with the variant CT and TT alleles exhibited lower sRAGE levels than those carrying the major CC genotype. Given the key role of sRAGE in neutralizing circulating proinflammatory RAGE ligands, it is conceivable that the individuals carrying the T allele at the rs2070600 variant express reduced levels of serum sRAGE, which may subsequently lead to an increase in RAGE-ligand interactions, thereby initiating cellular responses. Therefore, individuals with the T allele of the *RAGE* rs2070600 variant may be more susceptible to RAGE ligand-induced inflammatory responses, which is the leading cause of IS development.

Cerebral ischaemia-induced brain tissue injury activates several proinflammatory and cell death mechanisms that further increase cellular damage to the tissues at risk, which surround the infarct area, where activated microglia, infiltrated monocytes/macrophages and lymphocytes are present in high numbers [45]. In the acute phase after stroke, HMGB1 plays an important role in the induction of inflammation in ischaemic brain tissue mainly through microglial activation [16]. Blockade of HMGB1 signalling with short hairpin RNA in the post-ischaemic brain suppresses infarct size, microglial activation and the induction of proinflammatory mediators [18]. The use of anti-HMGB1 neutralizing antibodies in experimental models of MCAO/reperfusion led to a remarkable reduction in infarct size and an improvement in neurological deficits in treated rats [46]. These lines of evidence suggest that HMGB1 may contribute to the infarct size in IS. In the present study, we found that patients carrying the variant genotypes (CG and GG) of the *HMGB1* rs2249825 variant have lower infarct volumes than those carrying the major CC genotype. In MI patients, HMGB1 serum levels were significantly correlated with infarct size after MI [47]. It is conceivable that the levels of HMGB1 in patients carrying the G allele of the *HMGB1* rs2249825 variant are lower than that in patients with the major CC genotype, thereby contributing to lower infarct volumes. Therefore, rs2249825 variants might induce differential expression of HMGB1, which correlates with the infarct volume.

Our study has several limitations that should be accounted for when interpreting the results. First, as a retrospective case-control study, potential bias, including information bias, selection bias and confounding bias, cannot be entirely excluded. Second, only five variants of *RAGE* and four variants of *HMGB1* were evaluated in this study; other variants, especially low-penetrance loci and copy number variations, may also contribute to IS risk, and their combined effects should not be neglected for predicting the occurrence, severity and outcome of IS. Third, other clinical characteristics of the study group, such as the rates of hypertension, diabetes or hypercholesterolemia, may have masked the associations between the *RAGE* and *HMGB1* variants and IS. Fourth, the serum HMGB1 levels were not detected. Therefore, the effect of the rs2249825 variant on HMGB1 expression was not evaluated. The results obtained in this study require confirmation in independent studies involving larger populations with different ethnic backgrounds before the conclusions can be considered definitive and useful for estimating an individual’s risk of developing IS.

In conclusion, our study was the first to report that the variant G allele of the rs2249825 *HMGB1* variant plays a protective role in IS development. Our findings support the existence of an association between the rs2070600 variant of *RAGE* and the risk of developing IS in a southern Chinese population. Our study may provide clues for the evaluation of individual susceptibility to IS and for the development of effective measures for the control and prevention of IS. Additional studies are needed to shed light on the role of RAGE and HMGB1 in the pathogenesis of IS and to further clarify their prognostic and therapeutic potential.

## MATERIALS AND METHODS

### Participant recruitment

This hospital-based case-control study recruited 1,034 consecutive patients (688 male and 346 female) with IS from the Department of Neurology at the Affiliated Hospital of Guangdong Medical University between September 2012 and June 2015. An IS diagnosis was established based on the patient’s clinical signs and symptoms. All patients underwent magnetic resonance imaging and/or computed tomography scans as well as standard blood tests. All IS patients were classified into subtypes based on the Chinese IS subclassification (CISS) system [28] by two experienced neurologists. Patients with a history of transient ischaemic attacks, cardioembolism, cerebral haemorrhage, coronary artery diseases, autoimmune diseases, haematologic diseases, malignant tumours or chronic infectious diseases were excluded from the study. One subject diagnosed with recurrent stroke or stroke onset longer than 72 h was also excluded.

The control group consisted of 1,015 individuals (666 male and 349 female) who were recruited from the Health Examination Center of the Affiliated Hospital of Guangdong Medical University during the same period, and the controls were comparable to the IS subjects in terms of age and sex. In the control group, individuals with a recent history of cerebrovascular disease or myocardial infarction (MI) were excluded. A questionnaire was administered to both the case and control groups to assess risk factors. The information collected included demographic characteristics, medical history (hypertension, diabetes mellitus), daily cigarette smoking and parameters of hypercholesterolemia. Hypertension was defined as a systolic pressure of > 140 mm Hg and diastolic pressure of > 90 mm Hg on more than one occasion and/or the current use of antihypertensive drugs. Diabetes mellitus was defined as glucose levels of ≥ 7.0 mmol/L at fasting, ≥ 11.1 mmol/L 2 h after oral glucose challenge, or both, or receiving antidiabetic drugs. Subjects were considered smokers if they smoked more than 10 cigarettes per day for five years and drinkers if they drank more than 50 mL of alcoholic beverages per day for five years. Written informed consent was obtained from all of the enrolled participants, and this study was approved by the Ethics Committee of the Affiliated Hospital of Guangdong Medical University.

### Genotyping

Genomic DNA was isolated from human peripheral blood samples of each individual using the TIANamp Blood DNA kit (Tiangen Biotech, Beijing, China) according to the manufacturer’s instructions. The DNA concentration was determined using a DNA spectrophotometer (ND-1000, NanoDrop, Wilmington, DE, USA).

The five *RAGE* SNPs (rs1800625, rs1800624, rs2070600, rs1035798 and rs184003) and four *HMGB1* SNPs (rs1415125, rs2249825, rs3742305 and rs1045411) were selected based on previous studies [22, 23, 48]. Detailed information regarding the *RAGE* and *HMGB1* variants is shown in Supplementary Table 1 [51-54] and Supplementary Figure 1. The selected *RAGE* and *HMGB1* SNPs were genotyped using the SNaPshot Multiplex Kit (Applied Biosystems Co., Ltd., Foster City, CA, USA), and the primers for PCR amplification and SNaPshot extension were designed based on the GenBank database (Ref *RAGE* mRNA: NM_001206966.1 and Ref *HMGB1* mRNA: NM_002128.4) and are shown in Supplementary Table 2. The SNaPshot reactions and PCR procedures were performed as previously described [49].

### Enzyme-linked immunosorbent assay (ELISA)

Blood samples were collected as soon as the diagnosis was established. Blood specimens were drawn in EDTA-containing tubes and centrifuged at low speed, and the serum aliquots were stored at -20°C. The serum RAGE levels were determined in duplicate using the Quantikine sandwich ELISA kits (R&D Systems, Minneapolis, MN, USA) according to the manufacturer’s instructions.

### Infarct volume quantification

Infarct volumes indicated by diffusion-weighted magnetic resonance imaging (DWI) were measured with MIPAV software (Medical Image Processing, Analysis, and Visualization, version 3.0; NIH, Bethesda, MD) [50]. Acute diffusion lesions were defined on a slice-by-slice basis using a semiautomatic segmentation approach, consulting apparent diffusion coefficient and fluid-attenuated inversion recovery imaging sequences to distinguish acute from nonacute diffusion change. DWI infarct volumes were calculated by multiplying the slice thickness by the total lesion area.

### Statistical analysis

Statistical analyses were conducted using SPSS, version 19.0 (IBM, Armonk, NY, USA) and GraphPad Prism 4.0 (GraphPad Software, Inc., San Diego, CA, USA). Measurement data are represented as the mean ± standard deviation (SD) for continuous variables and as the median and percentage for quantitative variables; a chi-squared (*χ*^2^) test and Student’s *t*-test were used to compare variables between the two groups. The allele frequencies and genotype distribution of IS patients and controls were compared using the *χ*^2^ test or Fisher’s exact test. The odds ratio (OR) and 95% confidence interval (CI) were used as measures of the strength of an association between the *RAGE* and *HMGB1* genotypes and IS. The Hardy-Weinberg equilibrium (HWE), linkage disequilibrium (LD) and haplotypes were analysed using Haploview software package (version 4.2). Comparisons of the serum sRAGE levels among patients with different *RAGE* and *HMGB1* variants in the case and control groups were evaluated using Student’s *t*-test for normally distributed data, and for non-normally distributed data, a Mann-Whitney U nonparametric test was used. Correlations between the genotypes of the *RAGE* and *HMGB1* variants and the DWI infarct volume were assessed using analysis of variance (ANOVA). Linear regressions were adjusted for age, gender, smoking, hypertension, diabetes mellitus and hyperlipidaemia. Power calculations were performed with the program of Purcell *et al.* (available at http://zzz.bwh.harvard.edu/gpc/). Bonferroni correction was applied for multiple comparisons with control type 1 error. Gene-gene interactions were evaluated using Multifactor Dimensionality Reduction (MDR3.0.2). Statistical significance was set at P < 0.05 for all of the tests.

## SUPPLEMENTARY MATERIALS FIGURES AND TABLES




