# Supplementary material for: Genetic predisposition to ischaemic stroke by RAGE and HMGB1 gene variants in Chinese Han population
Source: Oncotarget. 2017 Oct 26;8(59):100150–64. doi: 10.18632/oncotarget.22112 (PMC5725009; doi:10.18632/oncotarget.22112)
Supplement: Supplementary file 1 [file oncotarget-08-100150-s001.pdf]

Genetic predisposition to ischaemic stroke by *RAGE* and *HMGB1* gene variants in Chinese Han population

SUPPLEMENTARY MATERIALS

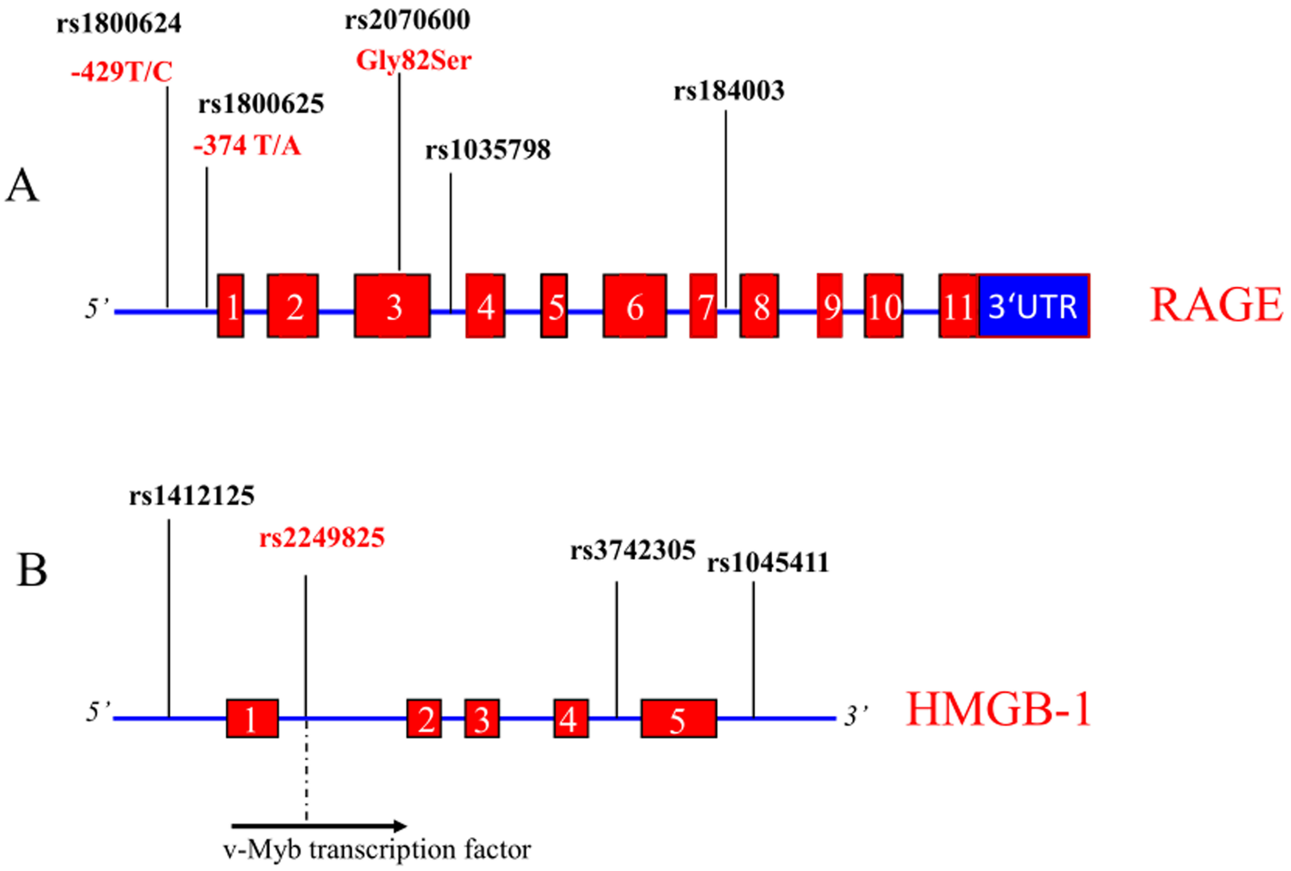

Supplementary Figure 1: Genetic structure of the *RAGE* **(A)** and *HMGB1* **(B)** genes. Exons are shown as red boxes (*RAGE*: exon 1-11, *HMGB1*: exon 1-5), and the promoter and intron sequences are shownas lines. Blue boxes represent the 3' untranslated regions. From the HapMap/Haploview database and the literature, 5 SNPs were identified in the *RAGE* gene (upper panel), and 4 SNPs were identified in the *HMGB1* gene (lower panel). Vertical lines indicate the locations of these polymorphisms. The arrow indicates the transcription factor.

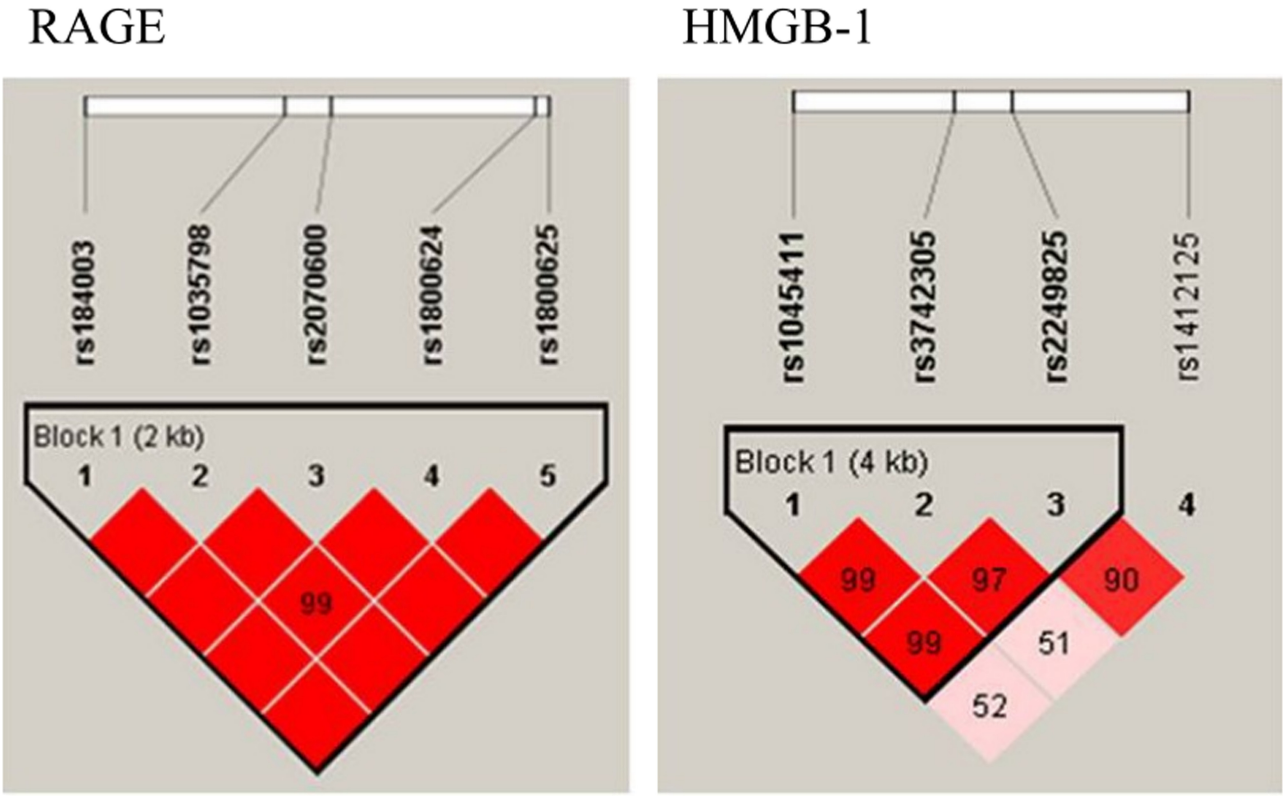

Supplementary Figure 2: The linkage patterns of polymorphisms in the *RAGE* and *HMGB1* genes. The shade of the diamonds represents the pairwise r^2 between the two SNPs as defined by the top left and top right sides of the diamond. Shading represents the magnitude of the pairwise r^2, with red shades reflecting high r^2 (>0.80) and white shades reflecting low r^2.

Supplementary Table 1 : Information on *RAGE* and *HMGB1* polymorphisms

Gene	rs number & mutation	position	predicted function	MAF			HWE
				HapMap -CHB	case	control	
RAGE	rs1800625/-429T/C	promoter	transcriptional regulation [30]	0.121	0.073	0.079	0.261
	rs1800624/-374T/A	promoter	transcriptional regulation [30]	0.163	0.126	0.133	0.254
	rs2070600/244C/T	exon3	N-glycosylation site [22]	0.237	0.209	0.182	0.066
	rs1035798/356G/A	intron3	splicing balance [51]	0.132	0.118	0.128	0.202
	rs184003/1704G/T	intron7	stability regulation [52]	0.168	0.166	0.183	0.518
HMGB1	rs1412125/-1615T/C	5'-flanking	enhancer [53]	0.241	0.312	0.329	0.401
	rs2249825/3814C/G	intron1	TF binding site [53]	0.111	0.151	0.176	0.926
	rs3742305/1177G/C	intron4	mRNA splicing or enhancer [54]	0.189	0.213	0.237	0.860
	rs1045411/6850G/A	3'UTR	miRNA binding site [53]	0.195	0.214	0.238	0.822

HapMap-CHB: https://www.ncbi.nlm.nih.gov/variation/tools/1000genomes/

Supplementary Table 2 : Primers used to genotype *RAGE* and *HMGB1* polymorphisms

Gene polymorphisms	Ref mRNA	Direction	Primers
RAGE			
rs1800624 & rs1800625	NM_001206966.1	forward	5'- GACCCACTGGAGCCCCATCT-3'
		reverse	5'-TTTTTCCCTGGGTTTAGTTGAGAATTT-3'
rs2070600	NM_001206966.1	forward	5'-GCTGGGGTTGAAGGCTTTTTCT-3'
		reverse	5'-CCGGACAGAAGCTTGGAAGGTC-3'
rs1035798	NM_001206966.1	forward	5'-TTCCCATCCAAGTGCCAGCTAA-3'
		reverse	5'- CCCATGAGGGCCTCTGACTTC-3'
rs184003	NM_001206966.1	forward	5'- GCCTTTCCCTCGTTAGCCCTCT-3'
		reverse	5'- CCTTCCAACTCCCAGAGCCTGT-3'
HMGB1			
rs1412125	NM_002128.4	forward	5'- TCCAACAACCAATTCCTCCAAATG-3'
		reverse	5'- GCATGTGTGATATATTGTCCATTCACC-3'
rs2249825	NM_002128.4	forward	5'- TGACATTTTGCCTCTCGGCTTCT-3'
		reverse	5'- GAGCAGACTCGGGCGGATAGAT-3'
rs3742305	NM_002128.4	forward	5'- TGGTCCTCTGCATTCTTTGAAGG-3'
		reverse	5'- GGCTGCGAAGCTGAAGGAAA-3'
rs1045411	NM_002128.4	forward	5'- TGTGATGGGACGATCATCAGAACT-3'
		reverse	5'- AATGGAAGTGGGAGGCAATTTAGA-3'

Supplementary Table 3 : Summary of the MDR analysis.

See Supplementary File 1
