# Supplementary material for: Genetic predisposition to ischaemic stroke by RAGE and HMGB1 gene variants in Chinese Han population
Source: Oncotarget. 2017 Oct 26;8(59):100150–64. doi: 10.18632/oncotarget.22112 (PMC5725009; doi:10.18632/oncotarget.22112)
Supplement: Supplementary file 2 [file oncotarget-08-100150-s002.doc]

Supplementary Table 3 : Summary of the MDR analysis

| Model | CV  consistency | Bal acc CV  testing | P  value |
| --- | --- | --- | --- |
| rs1800624 | 6/10 | 0.50 | 0.83 |
| rs2070600, rs1412125 | 6/10 | 0.50 | 0.95 |
| rs1800624, rs2070600, rs1412125 | 4/10 | 0.51 | 0.62 |
| rs1800624, rs2070600, rs1412125, rs1045411 | 6/10 | 0.52 | 0.17 |
| rs2070600, rs1412125, rs184003, rs1412125, rs3742305 | 9/10 | 0.51 | 0.38 |
| rs1800625, rs1800624, rs2070600, rs184003, rs1412125, rs3742305 | 10/10 | 0.51 | 0.38 |
| rs1800625, rs1800624, rs2070600, rs184003, rs1412125, rs2249825, rs3742305 | 10/10 | 0.51 | 0.17 |
| rs1800625, rs1800624, rs2070600, rs1035798, rs184003, rs1412125, rs2249825, rs3742305 | 6/10 | 0.51 | 0.17 |
| rs1800625, rs1800624, rs2070600, rs1035798, rs184003, rs1412125, rs2249825, rs3742305, rs1045411 | 10/10 | 0.51 | 0.17 |

Abbreviations: bal acc, balanced accuracy; CV, cross validation; MDR, Multifactor Dimensionality Reduction
